# Incomplete assessments: Including harms and trade-offs in assessing policy approaches to Covid-19

**DOI:** 10.1016/j.ssmph.2026.101983

**Published:** 2026-10-15

**Authors:** Itai Bavli, Brent Sutton, Kevin Bardosh, Sandro Galea

**Affiliations:** a W. Maurice Young Centre for Applied Ethics, School of Population and Public Health, University of British Columbia, Vancouver, BC, Canada; b Independent Researcher, Vancouver, BC, Canada; c Pandemic Evidence Collaboration, Kellogg College, University of Oxford, UK; d School of Public Health, University of Washington, USA

## Abstract

Policies to combat COVID-19 were often implemented without a systematic assessment of trade-offs with other public objectives. This approach implicitly assumed that these policies would do little harm to other aspects of public health. Recently published evaluations of pandemic-era policies have also largely avoided cost-benefit (or harm-benefit) analysis in public health decision-making. This is likely due to a narrow framing of their analyses to the possibility of underreaction to the pandemic. We argue that a disproportionate policy response framework can offer a valuable toolkit to understand the policy choices to combat the pandemic. This article first reviews The Lancet COVID-19 Commission and The BMJ COVID-19 series evaluating policies implemented during the pandemic in Canada, the United States, and the United Kingdom. We suggest that these documents, likely motivated by concern with underreacting to the pandemic, fall short in evaluating the trade-offs between the harms and benefits of COVID-19 policies to ensure that they are of net benefit to the public. We further argue that the failure to address these trade-offs contributed to an erosion in public trust in health authorities and the unequal impacts of the global COVID-19 response, especially among low-income countries.

## Highlights

• Measures to promote public health must consider both their harms and benefits.
• Recent evaluations of public interventions to control covid have failed to do so.
• Narrow framing of evaluations to the possibility of underreaction is the problem.
• Failure to consider the harms of public interventions has eroded trust.
• Public health authorities must consider trade-offs in policymaking and evaluation.

## 1 Introduction

The COVID-19 pandemic challenged public health and required timely responses despite incomplete evidence. In many respects, this was public health's finest hour, with the rapid development and deployment of effective COVID-19 vaccines. However, other measures to combat the pandemic have received much criticism – some warranted and some not – which has undermined trust in public health officials. This has prompted important discussions on how to weigh harms against benefits in policy decisions during public health emergencies, highlighting the need for a systematic identification and evaluation of trade-offs (Bollyky et al., 2023; Dupont & Galea, 2022; Gandhi et al., 2024; Green & Venkataramani, 2022; Kampf & Kulldorff, 2021).

Public health scholars have largely avoided employing a benefit-harm (or cost-benefit) framework for Covid. Morally, this is understandable because this often involves assigning a monetary value to life. However, in a world of limited resources, our natural reluctance to systematically assess benefits and harms impedes sound policy analysis. The use of cost-benefit analysis is common in other fields, such as environmental, consumer and financial regulation, areas that also impact the well-being of large populations. To ensure the adoption of appropriate policies in the future, and to restore trust in public health authorities, such as public health agencies and ministries/departments of health, might do well to acknowledge that interventions can cause harms (even if inadvertent) and to assess these harms against the putative benefits of public health interventions.

Assessing policy trade-offs is a well-established foundational principle in economics (Green & Venkataramani, 2022; Tabarrok, 2024). In part this is due to resource limitations: policy priorities must be set because governments do not have sufficient funds to pursue them all. The need to assess policy trade-offs also arises because the pursuit of one policy may inadvertently undermine others, what economists refer to as “negative externalities.” Of particular importance here are distributional impacts, where a given policy may place a higher burden on some socio-economic groups over others (Bavli et al., 2020; Glover et al., 2020; Green & Venkataramani, 2022; Aiseadha et al., 2023). Central to this approach is the notion that policy trade-offs exist and must be evaluated to maximize the public good (Tabarrok, 2024).

Public health^1^ would benefit from incorporating economic insights on policy trade-offs into decision-making processes (Tabarrok, 2024). Public health decisions often have multiple societal impacts, both positive and negative, making the prioritization of policy choices necessary (Angeli et al., 2021; Dupont & Galea, 2022). The nature and magnitude of these trade-offs vary across interventions; for example, school closures, lockdowns, and vaccine mandates each involve distinct ethical, social, and economic considerations that represent different trade-offs. Further, large-scale and restrictive interventions—such as lockdowns, curfew, school closures, and travel restrictions—are more likely to generate unintended consequences, given their broad and often uneven social, economic, and health impacts across populations.

Evaluating policy trade-offs during a pandemic, where both the virus and public health responses have complex short- and long-term effects on health and beyond is a difficult task (Bardosh, 2023; Bavli et al., 2020; Aiseadha et al., 2023). Lockdowns, for example, may slow virus spread and save lives, but carry substantial short- and long-term health and economic costs (Bardosh, 2023; Bardosh et al., 2022; Aiseadha et al., 2023), may increase mistrust (Schüklenk, 2023) and can exacerbate health and economic inequities (Glover et al., 2020; Schüklenk, 2023). Further, these impacts are often interconnected: restricting personal liberty can undermine trust in public health authorities, which in turn affects long-term health outcomes by undermining support for other health measures (Bardosh et al., 2022; Bollyky et al., 2023; Dupont & Galea, 2022). Economic conditions are also closely tied to health outcomes (Braveman & Gottlieb, 2014). Finally, policy goals, and the interpretation of scientific evidence backing interventions to support those goals, depend on the weight given to different societal values—such as efficiency, utility, equity, or liberty (Angeli et al., 2021; Dupont & Galea, 2022; Aiseadha et al., 2023). Preferred policy choices may therefore differ depending on the societal value selected (Schüklenk, 2023). This makes the evaluation of such policies particularly challenging, as decision-makers must balance the goal of preserving life with other societal objectives under conditions of uncertainty.

Given these complexities, how can we assess complex policy trade-offs associated with public health action? There is no simple answer to this question. However, this difficulty should not deter trade-off assessments being incorporated into both decision-making processes and assessments. Fortunately, the importance of examining policy trade-offs is gaining some attention from scholars studying pandemic responses (Angeli et al., 2021; Bollyky et al., 2023; Dupont & Galea, 2022; Gandhi et al., 2024; Green & Venkataramani, 2022; Jack et al., 2023; Tabarrok, 2025). Yet, despite this recent attention, policy trade-offs have too often been overlooked in pandemic scholarship and public discourse, particularly when assessing the overall success of national responses to COVID-19 approaches. This article seeks to highlight this gap.

To address this gap, we draw on policy literature that conceptualizes proportionality in government action and policy failure (Bovens & Hart, 1996; Fotaki, 2010; Gupta, 2001; Hammer et al., 2007; Hogwood & Gunn, 1984; Howlett, 2012; Hudson et al., 2019; Maor, 2012; McConnell, 2010, 2015; Moran, 2001; Mueller, 2020; Wallinga et al., 2015). In particular, we focus on a disproportionate policy perspective, which argues that governments can over- and under-react to situations requiring a policy response (Maor, 2025). Overreaction occurs when policies' net cost exceed their benefit (i.e., where the scale, stringency, or breadth of intervention is disproportionate to the problem) and underreaction when policies’ net benefit is lower than the relevant status-quo counterfactual (i.e., weaker or narrower than warranted by the risk) (Daugbjerg & McConnell, 2021; Hafsi & Baba, 2023; Maor, 2012, 2014, 2021, 2025; Shafi & Mallinson, 2023).

Based on this literature, government can act “too much” to achieve a certain goal or “not enough,” both of which are undesirable. Insights from the agency failure literature point to why this may occur (Carpenter, 2010; Carpenter & Ting, 2005, 2007; Heimann, 1993, 1997). One is due to errors of commission, in which public authorities adopt and implement the wrong policy. A second is errors of omission, where authorities fail to act when action is warranted. The notion of errors of commission and omission is relevant to public health because public health interventions can create harms (Bonell et al., 2015; Lorenc & Oliver, 2014) by taking excessive steps (error of commission) or by not doing enough to protect the public (error of omission) (Bavli, 2023; De Ville & Novick, 2010; Holtgrave, 2010).

Read together, these frameworks make both excesses and inaction visible: they help evaluate not only whether governments acted, but whether they acted appropriately relative to problem scale and context. We use this lens to evaluate prominent post-hoc reviews of national COVID-19 responses.

In the first part of the article, we show that efforts to evaluate the impact of national COVID-19 responses published by two leading medical journals—The Lancet and The BMJ—largely failed to consider policy trade-offs. This is a particularly notable oversight because these journals have published some of the most important studies on COVID-19, greatly aiding global policymaking. Next, based on the policy over- and under-reaction, we argue that evaluating the effectiveness of COVID-19 public health responses must consider both the harms and benefits of these policies to ensure that they are of net benefit to the public. We argue that the failure to address these trade-offs eroded public trust in health authorities. We also highlight the need to evaluate the global COVID-19 response beyond vaccine inequity among low- and high-income countries, assessing its unequal impacts. In the final section, we discuss the key implications of incorporating trade-offs into public health decision-making during emergencies, with a focus on public health policymaking and research.

## 2 The Lancet and BMJ covid inquiries

Discussions of policy approaches to COVID-19 have led to several official inquiries in high-income countries evaluating the effectiveness of public responses to the pandemic (Covid Inquiry, 2026; U.S. House of Representatives, 2024). The scientific community has also launched multiple reviews to assess and learn from COVID-19 policies, with prominent inquiries published between 2022 and 2024 by two leading medical journals: The Lancet and The BMJ. These journals played a critical role in informing policymakers during the pandemic, providing updates on the state of knowledge (e.g., the risk of the virus) and evaluating the effectiveness of non-pharmaceutical interventions (NPIs) like mask mandates and lockdowns.^2^

The Lancet Commission on lessons for the future from the COVID-19 pandemic (https://www.thelancet.com/commissions/covid19) (Sachs et al., 2022; The Lancet, 2022), published in 2022, provides a detailed analysis and assessment of government responses to COVID-19, plus recommendations on how future pandemics could be handled better. The BMJ reports include The *BMJ* series to inform the UK's COVID-19 Inquiry (https://www.bmj.com/covid-inquiry), accountability for Canada's COVID-19 response (https://www.bmj.com/canada-covid-series), and the US COVID-19 lessons for future health protection and preparedness (https://www.bmj.com/collections/us-covid-series). They encompass 25 analyses, opinions, and editorials, published between 2022 and 2024 (see Appendix 1), that address a wide range of topics (see Table 1). All articles published as part of the four analyses (the Lancet Commission and the three BMJ COVID-19 series) were included, as they collectively represent these journals' retrospective assessments of COVID-19 policy responses. We systematically reviewed each article, identifying instances in which authors discussed the potential harmful effects of public health interventions during COVID-19 or conducted trade-offs or cost–benefit assessments.

**Table 1:** Topics covered and reference to harm and trade-offs.

	Number of articles	Key topics covered	References to harm	Reference to trade-offs
The Lancet Commission (2022)	1	Comprehensive elevation of national COVID responses, with a particular emphasis on international cooperation	The report discusses harms only in a few sections, focusing briefly on the effects of measures on vulnerable populations: marginalized individuals, the poor, displaced children, and refugees. These discussions address impacts such as mental health issues, domestic violence, limited access to essential services, school closures, child abuse, global poverty, food insecurity, gender-based violence, and disruptions to immunization programs (Sachs et al., 2022; The Lancet, 2022).	Minor reference to cost-benefit considerations, but does not discuss them in detail.
BMJ United Kingdom series (2022)	9	Failure to use evidence, knowledge mobilisation, flawed policy response, policy on children and schools, mathematics models, learning networks in the pandemic, disinformation	Two articles (Gurdasani et al., 2022; McKee et al., 2022) provide a short reference to possible harm to children resulting from learning loss, but did not discuss these harms.	None.
BMJ Canada series (2023)	7	Global vaccine equity, effect on indigenous communities, decision making, failure to protect long-care care homes, health inequalities, misuse of research	One article (Estabrooks et al., 2023) provides a short reference to possible negative effects of restrictive measure on people living in long term care homes, but did not discuss them.	None.
BMJ United States series (2024)	9	Communication failures, structural racism, preparedness for next pandemic, Covid-19 in jails and prisons, weakness of US emergencies powers, US workers during Covid-19, US leadership in global health, harm reduction, global health	One editorial (Yamey et al., 2024) indirectly raises concerns that prolonged closures of schools, parks, and playgrounds may have led to negative consequences. However, these issues are not further discussed in the editorial or in any of the other articles in the series.	None.

The general narrative of both inquiries is that governments could have done more (under-reacted) to protect the public. We applaud these efforts for their positive contribution to improving public health policies during emergencies and preparing for future crises.

We use the occasion of these reports, however, to highlight an omission from both inquiries and from the public health conversation at large in the aftermath of the pandemic: examining the harms, along with the benefits, of public health measures and evaluating policy trade-offs. While these publications amply and appropriately showcase the successes of public health during the pandemic, we suggest that this success does not obviate the observation that public health interventions involve trade-offs between benefits and harms. The field of harm-benefit (or cost-benefit) analysis is well-established in public health, and the notion that there are trade-offs in our actions between benefits and harms should be uncontroversial (Dupont & Galea, 2022). It is therefore both surprising—and perhaps indicative of some of the challenges public health has faced in dealing with the pandemic—that The Lancet and The BMJ inquiries omit almost entirely the inherent trade-offs of public health interventions to address the pandemic.

The Lancet Commission mentions harms in a few places but does not discuss them systematically. Further, the Lancet Commission makes only a minor and cursory reference to policy trade-offs and does not incorporate them in its recommendations (Table 1) (Sachs et al., 2022). The three BMJ series fail to address the cost and the unintended harms of public health interventions, and only one out of the seven Canadian articles and two out of the nine UK articles offer even minor references to possible negative effects. One of the nine US articles provides a short and indirect reference to these issues in the editorial of the series (Yamey et al., 2024) (Appendix 1).

These references to harm were extremely limited. For example, in the only Canadian article that mentioned harm (Estabrooks et al., 2023), just two sentences (38 words) address the issue (Appendix 1). In total, these 38 words out of 13,033 words across the seven Canadian articles amount to only 0.29% of the text. Similarly, the four sentences (101 words) in the editorial of the US COVID-19 series (Yamey et al., 2024) that indirectly describe harms represent an extremely small proportion of the overall text; 101 words out of 16,299 total words written across nine articles amount to just 0.62% of the content in the articles. For the UK COVID-19 series, references to harm are likewise minimal. In total, nine sentences (207 words) across the nine articles address harms, representing just 0.83% of the overall content (207 out of 25,003 words). Taken together, references to harm account for less than 1% of the content across the 25 articles BMJ COVID-19 series.^3^ None of The BMJ articles incorporate cost-benefit analysis (Table 1).

Building on these reports, we discuss why it is vital to conduct a comprehensive analysis that incorporates trade-offs in health—avoiding the often-narrow focus on the death and harms from COVID-19 alone. Our goal is not to contradict or challenge issues raised by these inquiries. Rather, we highlight what has been systematically missed from The Lancet and The BMJ reports: (1) more fully acknowledging the harms of public health interventions; and (2) assessing and incorporating trade-offs in health in government response to COVID-19.

## 3 Why harms should not be ignored

There are at least three reasons why it is important to discuss the harms along with the benefits of public health interventions, as well as trade-offs in analyses of the pandemic response: (1) harms are real and should be weighed against benefits; (2) ignoring harms and trade-offs increases public mistrust of health authorities; and (3) the more harmful effects of non-pharmaceutical interventions (NPIs) in low-income countries. We discuss each in turn.

### 3.1 Public health policies created harms

Harms of public health interventions in the context of the pandemic were real, and increasingly known and documented (Bardosh et al., 2022; Bavli et al., 2020; Glover et al., 2020; Macedo & Lee, 2025, 2025hAiseadha et al., 2023). For example, a comprehensive framework has recently been published that identifies the range of harms of NPIs and reviews their magnitude (Bardosh, 2023). This farmwork also discuss the gaps in evidence and make suggestions for how to improve the academic research on public health harms. Another framework focuses on health inequalities, showing that several interventions not only perpetuated and exacerbated pre-existing inequities, but also created new ones (Glover et al., 2020).

As noted, none of The BMJ articles seriously address the harms of public health measures during COVID-19, and while The Lancet Commission discusses harms in some sections, it fails to do so comprehensively or to address them in its recommendations (see Table 1). Instead, the three retrospective BMJ analyses echo the dominant narrative early in the pandemic that preventing the spread of the virus and “bending the curve” should be pursued at almost all cost. This narrative, amplified by popular media and social media actors (Galea, 2023), largely ignored public health trade-offs, instead advocating for an all-in approach to COVID-mitigation as the best and only acceptable public health response.

The tendency to ignore harm is not unique to COVID-19. Public health has historically focused on prevention and has not invested the necessary resources to assess the negative consequences of public health choices (Bonell et al., 2015; Lorenc & Oliver, 2014). This tendency leads to policy assessments focusing mostly on failures to act. However, the harms of public health interventions can be significant and, in some cases, exceed their benefits (Bardosh et al., 2022; Bavli, 2023).

Policies to improve public health can disproportionately harm disadvantaged populations (Glover et al., 2020; Silva et al., 2013), contributing to unequal socio-economic outcomes (Sachs et al., 2022). Some populations are more susceptible to the coronavirus (e.g., older adults), while others might be more vulnerable to public health interventions: lockdowns disadvantage low-income populations and young people, social isolation measures harm those who struggle with addiction and mental health disorders, and school closures hurt children and their families (Bavli et al., 2020). Evidence from the US shows substantial learning losses associated with remote and hybrid schooling, with larger declines in districts with less in-person learning and in those serving more Black students; the authors note that these educational effects may be long lasting (Jack et al., 2023). Omitting harm from the analysis prevents us from examining how COVID-19 policies affect the well-being of different populations and potentially increase health inequities.

Discussion of harms by The Lancet Commission could have easily led to a harm-benefit assessment and included measures to limit harms in its recommendations. For example, disruption of immunization programs, and greater domestic violence, global poverty and food insecurity (Sachs et al., 2022), could have been weighed against the benefit of the policies that brought these about, and could have been incorporated in the report's recommendations. Referring to known harms of the pandemic response but failing to address them in the recommendations for preparedness for future pandemics undermines their importance at best and leaves policymakers with a flawed toolkit at worse.

Similarly, harms and trade-offs could have been easily incorporated into the articles in The BMJ reports in question. For example, briefly acknowledging inequality in learning loss in some UK articles (Gurdasani et al., 2022; McKee et al., 2022) could have led authors to focus on trade-offs in health, assessing the harms and benefits of school closures (Jack et al., 2023). Further, writing on the vulnerability of marginalized communities to the virus due to underlying disproportionate burden of poor health (Anderson & Mackinnon, 2023; Bentley-Edwards et al., 2024; Estabrooks et al., 2023; Feldman & Bassett, 2024; Michaels et al., 2024; Yamey et al., 2024) could have easily incorporated the effects of lockdowns on these same vulnerable populations. Similarly, articles focusing on global equity, including vaccine equity (Houston et al., 2023; Lee, 2023; Morrison & Gayle, 2024), could have referenced the unequal negative effects of NPIs designed in high-income countries to fight the virus in low-income countries.

### 3.2 Public trust in health authorities

Ignoring harms and trade-offs in Covid accountability investigations erodes public trust in health authorities (Giubilini et al., 2023). The history of epidemics teaches us that overly paternalistic public health interventions can lead to conflict and mistrust. As historian of epidemics, David Jones (2020), noted: “This step [epidemic response such as quarantine or compulsory vaccination] generally involves people with power and privilege imposing interventions on people without power or privilege, a dynamic that fuels social conflict.” Trust is a critical component in public health as it facilitates consent, a necessary condition to ensure the widespread adoption of public health measures, such as immunization programs. Lack of public trust increases non-compliance and vocal (and even hostile) opposition to actions taken by health authorities to promote public health (Bardosh et al., 2022; Bollyky et al., 2023). Further, marginalized, disabled, and vulnerable populations suffered disproportionately from lockdowns, social isolation measures, and school closures (Bavli et al., 2020; Glover et al., 2020). Excluding their experiences —or treating them as unimportant or, worse, mischaracterizing them in pejorative terms —could further increase mistrust and hostility toward health authorities. (Bollyky et al., 2023). This suggests that, in some contexts, less restrictive approaches may deserve greater consideration, especially given the mistrust that stricter measures may produce.

The erosion of trust in public health institutions may have negative consequences for public health. For example, in the United States and parts of Canada, routine childhood vaccination requirements are increasingly being challenged, vaccination exemptions are rising, and outbreaks of preventable diseases such as measles have re-emerged. These developments highlight the need for further research on the relationship between pandemic response, trust in public health institutions, and compliance with other essential public health programs (Bardosh et al., 2022).

### 3.3 Low-income countries and the unequal global response to COVID-19

The experiences of low-income countries are included in our analysis because both The Lancet Commission and the three BMJ series address the global responses to the pandemic. This focus is important, as the global response to the pandemic has been inadequate, leading to unequal socio-economic effects across countries (UNICEF, 2021). Data from the World Bank and UN agencies showed that over 400 million people fell below their national poverty line in 2022, with low-income countries experiencing a disproportionate share of the drop (World Bank, 2022). UNICEF estimates a 13% global increase in learning poverty (defined as the share of children who cannot read a simple text with comprehension by age 10), disproportionately higher in low- and middle-income countries, essentially erasing all educational gains since 2000, and estimated to cost the current generation of students $21 trillion in lost earning potential (World Bank Group, 2022). UNICEF further found that children's diets have deteriorated, and school closures imposed greater harms on children in low-income nations (UNICEF, 2021). Additionally, millions of children missed out on essential vaccines, with an increase of nearly four million unvaccinated children from 2019, and the disparity in recovery efforts has widened the gap between high and low income countries (UNICEF, 2021). Finally, low-income countries were less able to fund expanded welfare programs to deal with the economic harms of NPIs or support the debt associated with the increased spending that was undertaken.

More specific country-level evidence illustrates how pandemic restrictions could have especially severe effects in low-income settings, where large informal economies and constrained access to remote schooling reduced people's ability to absorb prolonged disruptions (Adesina et al., 2026). In Uganda, qualitative studies reported that lockdown measures were associated with disruptions to family stability, housing, caring responsibilities, children's welfare, schooling, food security, and diet, as well as increased gender-based violence, child labour, sexual exploitation, and early marriage; these studies also reported livelihood losses, reduced household income, business closures, and heightened poverty risks (Adesina et al., 2026; Musoke, Deane, et al., 2024; Musoke, Nalinya, et al., 2024); in Angola, where 77% of the population was active in the informal economy, unemployment in September 2020 was 34% higher than in 2019, youth unemployment reached 56.4%, and a survey of 48 informal-sector operators in Luanda found that 47.9% reported reduced working days, 22.9% a total stoppage of activity, 95.8% either significant or marginal reductions in income, and 41.7% reduced food expenditure (UNCTAD, 2022); In India, the March 2020 nationwide lockdown and suspension of transport links abruptly disrupted migrant workers' livelihoods, leaving many with few resources and prompting large numbers to return to their home states (Irudaya Rajan et al., 2020).

The Lancet Commission addressed this issue by advocating for the establishment of a Global Health Fund for low- and middle-income countries (LMICs), and by emphasizing the importance of LMIC representation and leadership. In contrast, BMJ discussions of the impact of national pandemic policies on low-income countries are limited to vaccine inequity. Further, The Lancet's call for greater international cooperation and coordination in the response to emerging infectious diseases partially addresses power imbalances in global health, with the call for more leadership in these efforts from LMICs is a good start. Acknowledging that the global response to the virus may have contributed to poverty in low-income countries (Adesina et al., 2026; UNICEF, 2021; World Bank Group, 2022) can allow a cost-benefit reexaminations of these policies which will inform future pandemics' response.

Hence, we suggest that any investigation examining the global response must critically evaluate the trade-offs in health within specific national contexts. Low-income countries, which often have younger populations at lower risk of serious harm from Covid-19, and who lack necessities like clean water and food, may benefit from less restrictive approaches (ÓhAiseadha et al., 2023). These countries lack the fiscal capacity to offset the economic harms of NPIs. The most appropriate national policies are conditional on national demographic, social and economic conditions, and any credible assessment of the pandemic response at the global level must take these factors into consideration.

## 4 Disproportionate policy responses and Covid-19 inquiries

The disproportionate policy response literature (Maor, 2025) teaches us an important lesson: governments can either take excessive action to address a problem or fail to act sufficiently. In both cases, the outcome is suboptimal or even harmful. This framework is useful for assessing the successes and failures of policies during the pandemic. Its central analytical contribution is the concept of proportionality: whether the intensity, timing, and scope of a policy response are justified relative to the scale of the problem, the available evidence, and the policy's broader consequences. By applying this framework, we can evaluate if policies may have gone “too far,” e.g., creating harms greater than their benefits, or if policies were not taken—or taken too late—to adequately protect the public.^4^

The disproportionate policy response framework highlights the existence and variety of disproportionate policy responses and trade-offs, and offers a useful lens for retrospectively analysing complex policy choices such as those adopted during the pandemic. It allows for a more nuanced evaluation of the successes and failures of government responses to public health emergencies. By examining the possibilities of both under- and over-reaction, this framework may prevent unintended biases to both possibilities. It forces the analyst to consider counterfactual impacts across a broad range of policy options. Applying this framework to the evaluation of the pandemic response can therefore play an important role in improving future policy responses to public health emergencies.

The BMJ and Lancet focused almost entirely—though without explicitly using the term—on the policy under-reaction perspective, while largely ignoring the possibility of policy over-reaction. This narrow framing prevents a thorough investigation of trade-offs, as the analysis highlights failures to act when action was needed while celebrating actions deemed successful. To ensure the adoption of appropriate policies in the future, and to restore trust in public health authorities, we must be willing to acknowledge that some public health interventions cause harms (even if inadvertent) and to assess these harms against the putative benefits of public health interventions. The disproportionate policy perspective provides a useful starting point for a fulsome evaluation of policy trade-offs during the pandemic.

This perspective is further supported by a recent analysis that seek to explain why policy overreaction may have occurred, and under what conditions such responses become more likely. Macedo and Lee (2025), for example, offer several reasons why policy decision-makers over-reacted to the Covid pandemic. First, national committees advising governments on policies to combat the crisis were dominated by public health officials, who neither had the expertise to evaluate the cost of NPIs nor the inclination to do so. These public health officials freely admit that reducing mortality was their primary concern and that any negative impacts of their recommendations was outside their expertise or concern. As Francis Collins, a member of the White House Coronavirus Task Force, commented, “You attach infinite value to stopping the disease and saving a life. You attach zero value to whether this actually totally disrupts people's lives, ruins the economy, and has many kids kept out of school …” (cited in Macedo & Lee, 2025).

Second, the media failed to ask the right questions. They were too focused on disease spread and deaths, and not enough about the costs associated with measures to prevent their occurrence. Third, academics also failed to question the costs or the efficacy of NPIs. Incentives played a role here. As Macedo and Lee (2025) note, “Scholars willing to investigate trade-offs involved in pandemic restrictions often found themselves subjected to invective, character attacks, and reputational damage …“.

More generally, the political science literature on comparative public policy has identified several conditions where significant policy change is likely to take place, though it is important to note that these are only necessary conditions since not all major policy changes are overreactions. One condition is issue salience, which is the degree to which the public is aware of an issue (Culpepper, 2011; Vogel, 1993). The more the public is paying attention and demanding action, the more likely a government will institute meaningful policy change. This factor is especially strong during crises, when the public feels anxious and vulnerable (Vogel, 2003). This captured public sentiment during the pandemic when the public had little else on their mind.

Another condition supporting major policy change is a low number of veto players (Immergut, 1990; Tsebelis, 1995). A veto player is a person, group or institution who must consent to a policy change. Veto players include heads of government, the executive branch (including administrative agencies), legislatures, judiciaries and societal groups with a key stake in the decision. Major policy change is more likely when decision-making authority rests with a single veto player. During the pandemic, major policy decisions were taken almost exclusively by the executive branches, which were insulated from other branches of government. This allowed for the quick adoption of far-reaching measures to limit the spread of the diseases, but came at the cost of meaningful oversight from other government bodies.

## 5 Conclusion

We examined The Lancet COVID-19 Commission and The BMJ COVID-19 series evaluating policies implemented during COVID in Canada, the United States and the United Kingdom. We found very limited structured trade-off evaluations within these assessments and, in particular, that the *now* known harms of public health responses were largely ignored. This is likely due to a narrow framing of their analyses to the possibility of underreaction to the pandemic. We argue that disproportionate policy response framework offers a valuable toolkit for assessing policies during public health crises, such as the COVID-19 pandemic, by helping to identify critical trade-offs that protect the public and strengthen preparedness for future emergencies.

The need to address trade-offs should be made explicit in the terms of reference of any inquiry. Further, invitees to hearings should reflect a broad range of views, which would place pressure on committee members to consider the appropriate balance in their recommendations. To be blunt, the final report of any public inquiry is shaped to a large degree by the questions that are asked, and the issue of policy trade-offs must be made explicit in their terms of reference. The fact that The Lancet and The BMJ inquires largely ignored policy trade-offs is discouraging. Diversity of thought, free from ideological bias, is key to assessing the adequacy of government responses to the pandemic. To improve public health outcomes, to prevent the recurrence of policy mistakes, and to restore trust in public health authorities, accountability inquiries must assess the harms as well as the benefits from public health interventions and weigh them against each other. So far, this has not been done.

## CRediT authorship contribution statement

**Itai Bavli:** Writing – review & editing, Writing – original draft, Methodology, Investigation, Conceptualization. **Brent Sutton:** Writing – review & editing, Investigation, Formal analysis, Conceptualization. **Kevin Bardosh:** Writing – review & editing, Methodology, Investigation, Formal analysis, Conceptualization. **Sandro Galea:** Writing – review & editing, Methodology, Investigation, Formal analysis, Conceptualization.

## Declaration of competing interest

The authors declare the following financial interests/personal relationships which may be considered as potential competing interests:None. If there are other authors, they declare that they have no known competing financial interests or personal relationships that could have appeared to influence the work reported in this paper.

## Data Availability

No data was used for the research described in the article.

## Footnotes

1 We adopt a broad definition of public health that includes the wider health, social, and economic determinants of population health.

2 Non-pharmaceutical interventions (NPIs) in the context of COVID-19 refer to public health measures implemented to reduce transmission of the virus. They act by reducing the rate of contact between individuals and by lowering the probability of transmission when contact occurs. These interventions include case detection and isolation (e.g., home isolation of infected individuals), contact tracing and quarantine of exposed individuals, travel restrictions, and restrictive closures such as gathering limits, school closures, and non-essential business closures. They also include curfews and personal protective measures such as physical distancing and mask-wearing (Etemad et al., 2023; Rees et al., 2022).

3 Word counts for all three series exclude references, boxes, images, and tables.

4 Political economy perspectives (Shughart, 2011; Tabarrok, 2025) emphasize that preparedness and responses to emergencies are often shaped by political incentives, including fear of blame (Hood, 2011), reelection considerations, and a focus on short-term outcomes (Jacobs, 2011).
