# Supplementary material for: Incomplete assessments: Including harms and trade-offs in assessing policy approaches to Covid-19
Source: SSM Popul Health. 2026 Oct 15;36:101983. doi: 10.1016/j.ssmph.2026.101983 (PMC13696212; doi:10.1016/j.ssmph.2026.101983)
Supplement: Supplementary file 1 [file mmc1.docx]

**Appendix 1: All identified references to harms in the BMJ COVID-19 series (UK, Canada, and the US)**

| **Articles reviewed** | **All identified quotations referring to harms** |
| --- | --- |
| **The *BMJ* series to inform the UK’s COVID-19 Inquiry (2022):**  1. Effective knowledge mobilisation: creating environments for quick generation, dissemination, and use of evidence (Ahmad, Gordon, Aylin, Redhead, & Holmes, 2022)  2. How covid-19 spreads: narratives, counter narratives, and social dramas (Greenhalgh, Ozbilgin, & Tomlinson, 2022).  3. Covid-19 in the UK: Policy on children and schools (Gurdasani et al., 2022).  4. Role of scientific advice in covid-19 policy (Jarman, Rozenblum, Falkenbach, Rockwell, & Greer, 2022).  5. Implementation of covid-19 vaccination in the United Kingdom (Majeed, Pollock, Hodes, & Papaluca, 2022).  6. Guided by the science? Questions for the UK’s covid-19 public inquiry (McKee, Hanson, & Abbasi, 2022).  7. Role of mathematical modelling in future pandemic response policy (Pagel & Yates, 2022).  8. Learning networks in the pandemic: mobilising evidence for  Improvement (Sidhu, Ford, Fulop, & Roberts, 2022).  9. Understanding and neutralising covid-19 misinformation and  Disinformation (Wang et al., 2022). | “Children in the United Kingdom have been severely affected by the covid-19 pandemic. The closure of schools deprived them of access not only to education but to the many other things that schools provide, from emotional support and life skills to, for some, regular meals. Some schools, especially those attended by children from more affluent families, were able to partially compensate by moving lessons online, but many could not, not least because many of their pupils were in families that were digitally excluded. Children from disadvantaged families were also disproportionately affected by bereavement as many had breadwinners in jobs that placed them at high risk of infection” (Gurdasani et al., 2022).  “Lack of appropriate remote schooling provision and technological barriers affected children unequally.99 The most deprived students and students in state schools and colleges were less likely to experience online learning and have interactions with teachers, students, and peers than less deprived students and students in independent schools.99 Inequalities in loss of learning in reading and numeracy predictably continued well into 2021 because of lack of support, particularly for disadvantaged students. Stripping back of catch-up funding for children has left schools, children, and families struggling” (Gurdasani et al., 2022).  “Children have been harmed through covid affecting them or family members, and through loss of education.”16 (McKee et al., 2022)* |
| **Accountability for Canada’s COVID-19 response (2023):**  1. We can learn from the covid-19 pandemic management strategy of First Nations communities in Canada (Anderson & Mackinnon, 2023).  2. How Canada’s decentralised covid-19 response affected public health  data and decision making (Bubela, Flood, McGrail, Straus, & Mishra, 2023)  3. The world expected more of Canada (Clark, Straus, Houston, & Abbasi, 2023).  4. The predictable crisis of covid-19 in Canada’s long term care homes (Estabrooks, Ewa, Keefe, & Straus, 2023).  5. Canada’s role in covid-19 global vaccine equity failures (Houston, Liu, Habibi, Murthy, & Pai, 2023).  6. Canada’s pandemic response and future preparedness (Lee, 2023).  7. Use and misuse of research: Canada’s response to covid-19 and its  health inequalities (Mishra et al., 2023). | “Initial restrictive measures were perceived to have devastating effects on residents by increasing loneliness, depressive symptoms, and behavioural problems, although no Canada-wide data are available.58 These concerns led to questions about the residents’ right to live at risk.59” (Estabrooks et al., 2023). |
| **The US COVID-19 lessons for future health protection and preparedness (2024):**  1. Race, racism, and covid-19 in the US: lessons not learnt (Bentley-Edwards, Adisa, Ruff, McClure, & Robinson, 2024).  2. Is the US prepared for the next pandemic? No, but it could be (Cipriano, Kadakia, & Chokshi, 2024).  3. US public health after covid-19: learning from the failures of the hollow  state and racial capitalism (Feldman & Bassett, 2024).  4. Covid-19 in US jails and prisons: implications for the next public health crisis (LeMasters & Brinkley-Rubinstein, 2024).  5. Legal infrastructure for pandemic response: lessons not learnt in the  US (Mello, Jiang, Platt, Moran-McCabe, & Burris, 2024).  6. US workers during the covid-19 pandemic: uneven risks, inadequate  protections, and predictable consequences (Michaels, Spieler, & Wagner, 2024).  7. After covid-19: the case for optimism for US leadership in global health (Morrison & Gayle, 2024).  8. Building harm reduction into global health security and pandemic  prevention (Popescu & Malatyrivera, 2024).  9. Pandemic lessons for the 2024 US presidential election (Yamey, Diez Roux, Clark, & Abbasi, 2024). | “The absence of timely evidence and delayed or incomplete communication of what was known also led to over-reach, which itself had harmful consequences. For example, even after studies had shown that fomite transmission was rare and transmission outdoors was much less common than indoors,22 23 some municipalities and states kept parks, playgrounds, and beaches closed. Even after research had shown that schools could be reopened safely with basic public health measures, too many jurisdictions kept teaching online only. Too often, there were failures to learn from evidence based harm reduction approaches that have been successful in curbing other pandemics, such as HIV/AIDS” (Yamey et al., 2024). |

* This is an editorial. The sentence cites the article by Gurdasani et al. (2022), referenced above.
